# The impact of preceding noise on the frequency tuning of rat auditory cortex neurons

**DOI:** 10.1186/1471-2202-13-70

**Published:** 2012-06-18

**Authors:** Yinting Peng, Pengpeng Xing, Juan He, Xinde Sun, Jiping Zhang

**Affiliations:** 1 Key Laboratory of Brain Functional Genomics, Ministry of Education, Shanghai Key Laboratory of Brain Functional Genomics, Institute of Cognitive Neuroscience, School of Life Science, East China Normal University, Shanghai, 200062, China

**Keywords:** Noise, Auditory cortex, Forward masking, Frequency tuning, Receptive field

## Abstract

**Background:**

In a natural environment, contextual noise frequently occurs with a signal sound for detection or discrimination in a temporal relation. However, the representation of sound frequency by auditory cortical neurons in a noisy environment is not fully understood. Therefore, the purpose of this study was to explore the impact of contextual noise on the cortical tuning to signal sound frequency in order to better understand the mechanism of cortical frequency coding in a complex acoustical environment.

**Results:**

We compared the excitatory frequency-level receptive fields (FLRFs) of neurons in the rat primary auditory cortex determined under both quiet and preceding noise conditions. Based on the changes of minimum threshold and the extent of FLRF of auditory cortical neurons, we found that the FLRFs of a cortical neuron were modulated dynamically by a varying preceding noise. When the interstimulus interval between noise and the probe tone was constant, the modulation of the FLRF increased as the level of noise was increased. If the preceding noise level was constant, the modulation decreased when the interstimulus interval was increased. Preceding noise sharpened the bandwidth of the FLRFs of 47.6% tested neurons. Moreover, preceding noise shifted the CFs of 47.6% neurons by more than 0.25 octaves, while the CFs of the rest of the neurons remained relatively unchanged.

**Conclusions:**

The results indicate that the cortical representation of sound frequency is dynamically modulated by contextual acoustical environment, and that there are cortical neurons whose characteristic frequencies were resistant to the interference of contextual noise.

## Background

In a natural environment, perceived sensory target stimuli often occur with stimulus contexts in a temporal relation. These contextual stimuli are either temporally overlapping or adjacent to the perceived target stimuli. Psychophysical studies have demonstrated that contextual stimuli can suppress the perception of a target stimulus in the auditory [1-3] and visual [4-6] system. Previous neurophysiological studies have demonstrated cortical neural processing of contextual stimuli in auditory cortex [7,8], visual cortex [9,10] and somatosensory cortex [11]. These findings suggest that the contextual masking effect on the response to a target stimulus is common in different sensory modalities.

In particular, the auditory scene is dynamically changing in time as sound sources enter and leave our space. Many studies have emphasized the importance of dynamic aspects of auditory information processing. For instance, in a dynamic acoustic environment, an effective preceding sound (i.e., masker) can elevate the detection threshold of a following target sound (i.e., probe). This forward masking effect has been demonstrated in both human [1,3] and animal [12] behavioral studies. A forward masker also affects the intensity discrimination [13] and frequency discrimination [14,15] of a probe. These studies demonstrated that the forward masking effect is dependent on the acoustical parameter (e.g., frequency, level, and time) of both the masker and the probe.

The neural correlation of forward masking in the auditory system has been investigated for several decades. It has been shown that a forward masker mainly had a suppressive effect on the responses of cortical neurons to a target stimulus [16-20]. Based on the parameter of the masker and target stimulus, the suppressive effect can last for several hundred milliseconds [8,21] or longer [7]. In contrast, several studies have reported facilitative effect showing enhanced response by a preceding sound in the auditory cortex [22-24]. These studies have increased our understanding regarding how the cortical neurons respond to sounds in sequence in a dynamic acoustic environment.

The receptive field of an auditory neuron reflects the extent that the neuron tunes itself to particular stimulus parameters. Since contextual stimuli typically alter the responses of auditory cortical neurons to an acoustical stimulus, the characterization of contextual modulation of the receptive field of a neuron is crucial to understanding the contextual interaction in auditory information processing in a natural environment. Studies in the auditory cortex have shown that a preceding sound can reduce the spatial receptive filed [25] and level receptive field [16] of cortical neurons; however, the preference of cortical neurons to space and level remained relatively stable. Previous studies regarding contextual acoustical frequency information processing were largely focused on the characterization of inhibitory frequency receptive field in order to probe the mechanism of lateral inhibition or forward suppression on frequency tuning [26-28]. These studies have focused on how a varying preceding sound affects the responses to a fixed probe in the excitatory frequency receptive field. In contrast, we recently found that the excitatory frequency receptive fields of rat cortical neurons were dynamically modulated by a varying preceding tone [29]. In a natural acoustical environment, noise frequently occurs with the signal sound of interest. However, it is not clear how contextual noise affects the frequency tuning of auditory cortical neurons. Therefore, the aim of the present study was to determine the impact of preceding noise on frequency receptive field of cortical neurons and provide data for our understanding of cortical tuning of sound frequency in a noisy environment.

## Methods

### Animals and surgery

Experiments were performed on 35 healthy adult Sprague–Dawley rats (8−10 weeks old, body weight 250−300 g) showing no signs of outer or middle ear pathology. The rats were purchased from Shanghai SLAC Laboratory Animal Co. LTD (Shanghai, China). Animal care and experimental protocol were approved by the Animal Care Guidelines of the East China Normal University, and were in accordance with the National Institute of Health Guide for the Care and Use of Laboratory Animals (NIH Publications No. 80–23, revised in 1996). All efforts were made to minimize the number of animals used and their suffering. Rats were anaesthetized before surgery with an intraperitoneal injection of sodium pentobarbital (40 mg/kg body weight), and the dosage was maintained throughout the experiment by continuous intraperitoneal infusion of sodium pentobarbital via an automatic microinfusion pump (WZ-50 C6, Zhejiang University Medical Instrument Co. LTD, China).

At the beginning of surgery, the rats also received a single subcutaneous dose of atropine sulfate (0.01 mg/kg body weight) to reduce bronchial secretions. The body temperature of the rats was monitored by a rectal probe and maintained at 37.5°C by utilizing a feedback-controlled heating blanket. Following tracheal cannulation, the dorsal and temporal skull was then exposed, and a nail (2 cm long) was attached to the dorsal surface of the skull with 502 super glue and dental cement. The head of the rat was fixed through the nail to a head holder that was attached to a stainless steel platform, and a craniotomy over the temporal cortex was performed. A small incision was made in the dura to expose a portion of primary auditory cortex (AI). Warm saline was applied to the cortex during the experiment to prevent drying.

### Recording system

Recording of the responses of cortical neurons to acoustical stimuli was conducted in a double-wall room that was sound-insulated. The inside walls and ceiling were covered with three inches of convoluted polyurethane foam to reduce echoes. Glass electrodes (1.5−2.0 MΩ impedence, filled with 3 M KCl) were advanced orthogonal to the pial surface of AI through a remotely controlled microdrive (SM-21, Narishige, Japan) from outside the sound-insulated room. The electrode signal was amplified (1000×) and filtered (0.3−3.0 kHz) by a DAM80 pre-amplifier (WPI, USA), and the signal was sent to a RZ-5 Bioamp data processor (Tucker-Davis Technologies, TDT3, USA). In addition, the signal was also monitored in parallel via a digital oscilloscope (TDS 2024, USA) and an audiospeaker.

### Acoustic stimuli

**Figure 1:**
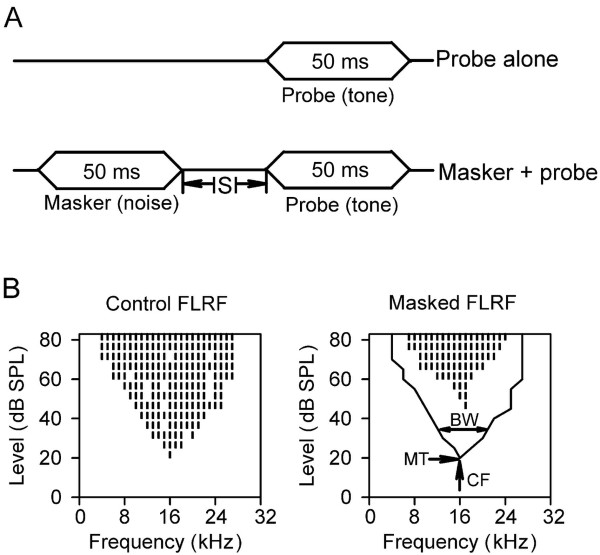
**An illustration of the stimulus paradigm and the construction of frequency-level receptive field (FLRF).A**) The probe alone paradigm was used to construct the control FLRF, and the forward masking paradigm (masker + probe) was used to construct the masked FLRF with preceding noise (masker). **B**) The left panel represents the control FLRF, and the right panel represents the masked FLRF under preceding noise conditions. The line curve in the right panel is the boundary of the control FLRF for comparison. Each short vertical line in the left and right panels represents a frequency versus level combination that elicited at least one spike for two presentations of probe stimuli. MT, minimum threshold; CF, characteristic frequency; BW, bandwidth of the FLRF. See texts in methods for details.

The acoustic stimulus generation and delivery system was a neurophysiology workstation (TDT3, USA) including a multifunction processor (RX6-A5), two programmable attenuators (PA5), an electrostatic speaker driver (ED1), and a free field electrostatic speaker (ES1). The sound stimuli used in the present study were binaural and free-field acoustical stimuli. The speaker was located 20 cm from the middle point of interaural axis of the rat, and was positioned at 45 degree in azimuth and 0 degree in elevation in the frontal auditory space contralateral to the hemisphere of the cortex being studied. The speaker was calibrated from 4.0 kHz to 44.0 kHz (sampling rate, 100 kHz) under computer control with a 0.25-inch condenser microphone (model 7016, ACO Pacific Inc.) that was placed at the animal’s ears. The calibrations were stored in the computer for use in controlling attenuators to obtain the desired sound pressure levels in decibel (dB SPLs) within the calibrated frequency range. The sound pressure level varied within ± 2 dB between 4 to 44 kHz. A forward masking paradigm was used in the present study (Figure 1A). The probe stimulus was a tonal stimulus, and the masker stimulus was a preceding noise. The preceding noise was a broadband white noise (the frequency spectrum ranged from 4.0 to 40.0 kHz), The duration of the masker and the probe was 50 ms with a 5 ms rise-fall time. For each trial of stimuli, the masker and probe stimuli were presented with an interstimulus interval (ISI) between the masker offset and the probe onset (Figure 1A). The presentation interval between two successive trials of acoustical stimuli was 800 ms. In the present study, the independent variables of the acoustic stimuli included the level of the masker, the frequency of the probe, the level of the probe, and the ISI between the masker and the probe.

### Data collection and analysis

For each rat, some preliminary electrode penetrations were made to get a coarse tonotopic map in the auditory cortex to identify AI. AI was defined in this study based on physiological properties such as short-latency response and continuous tonotopy (i.e., the CF of neurons increases from posterior to anterior) [30]. Single neurons were then isolated along tonotopic axis of the rat AI. Once a single neuron was well isolated in the third and fourth layers (estimated from the recording depth of penetration) of AI, a coarse range of sound frequency and level at which the neuron responded was first delimited audio-visually based on a criteria of 50% firing probability. The frequency-level receptive field (FLRF) of the neuron was then determined by using a stimulus matrix (frequency vs. level) presented with 1 kHz step in frequency and 5 dB steps in level. This stimulus matrix was always larger than the audio-visually determined frequency vs. level range. If the frequency tuning of the neuron was very sharp, the frequency step was changed to 0.5 kHz.

In both quiet and forward masking conditions, we determined the FLRF of a neuron based on the responses to two presentations per frequency level combination of probe stimuli. The response types of our isolated neurons to noise and tonal stimuli were either “phasic on” or “phasic burst on”, and the responses were within the noise or the tonal stimulus duration. Therefore, the responses to a probe stimulus were determined within the probe stimulus duration. Similarly, the responses to a masker stimulus were determined within the masker stimulus duration. The frequency level combination that elicited at least one spike in the two presentations of probe stimuli was used to construct the FLRF (Figure 1B). In probe alone condition (quiet condition, Figure 1A), the control FLRF was determined (Figure 1B, left panel), and the characteristic frequency (CF) and the tone minimum threshold (MT) were then determined from the FLRF online (Figure 1B, right panel). The tone MT of a neuron was the lowest stimulus level that evoked a response across the tested frequency ranges (i.e., 4.0 kHz to 44.0 kHz). The CF of a neuron was the frequency determined at its MT. If the FLRF was U shape, the CF of the neuron was defined as the middle frequency within the frequency range determined at MT. The noise rate-level function of a neuron was determined based on the responses to 30 trials of the broadband noise stimuli (masker only) from 0 to 80 dB SPL. The noise MT was defined as the noise level at which the response was 20% of the maximal response from the noise rate-level functions. A forward masking paradigm (Figure 1A) was then used to determine the FRLF under certain masker conditions (Figure 1B, right panel). The level of the masker was set at 10 to 40 dB above the noise MT so that the response of the neuron to a probe stimulus was significantly affected by the masker. To study the impact of the masker level on FLRF, the ISI was fixed whereas the level of the masker was varied. In this part of the experiment, for most neurons, the ISI between the masker offset and the probe onset was set to 50 ms; for few neurons, the ISI was set to a greater value (> 50 ms) if the masking effect of low level noise (at 10 dB above noise MT) at 50 ms ISI was strong enough to wipe out all the responses to the probe stimuli within the control FLRF. Likewise, to examine the temporal effect of the masker on the FLRF, the masker level was fixed at 20 dB above threshold whereas the ISI was varied. If the neuron was available for recording for several hours, both the ISI and the level of the masker were used as variables. In addition, a recovered FLRF in quiet conditions was determined whenever possible. A stable threshold at the CF of the neuron indicates a stable response of the neuron. A steady increase in threshold at CF was considered to be unstable in response and the neuron was excluded from the data analysis.

The MT shift, the area change of FLRF, the CF shift, and the change of bandwidth of FLRF were used as indices to analyse the impact of a preceding stimulus on the FLRF. The MT shift calculated as: MT in masked FLRF − MT in control FLRF. The area change of FLRF calculated as: [(area of masked FLRF − area of control FLRF)/area of control FLRF] × 100%. The CF shift defined as: CF under forward masking conditions − CF under control conditions. The CF shift was considered non-significant if it was not more than 0.25 octaves. The change of bandwidth of FLRF calculated as: [(bandwidth of masked FLRF − bandwidth of control FLRF)/bandwidth of control FLRF] × 100%. The bandwidth at n dB above MT (BWn) was categorized as widened if the change of bandwidth was greater than 20%, and was categorized as narrowed if the change of bandwidth was less than −20%. Otherwise, the bandwidth at n dB above MT was categorized as unchanged.

## Results

We determined the FLRFs of 57 neurons in the AI from 35 rats. Among these 57 neurons, the FLRFs of 42 neurons were determined when the noise levels were varied and the ISI was fixed. In addition, the FLRFs of 15 neurons were determined at various ISIs to study the temporal effect of the preceding noise on the FLRFs. Due to the time consuming process of recording, we presented the acoustic stimuli with a high-resolution of frequency and level combinations, and we minimized the repetition of stimuli (two repetitions/condition) at each frequency vs. level condition. Therefore, the detection of the effect of a forward masker on the FLRFs of an AI neuron relied on a change in MT, CF, area change of FLRF, and the bandwidth of FLRF.

### The effect of preceding noise on frequency tuning was level-dependent

**Figure 2:**
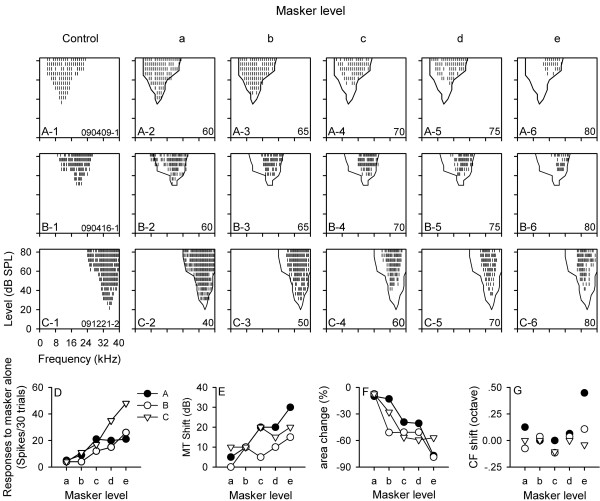
**Three representative neurons showing level-dependent modulation of frequency tuning by preceding noise (masker).** The FLRFs of each neuron under different acoustic conditions are shown in each row from rows 1 to 3. The panels in the first column are the control FLRFs of neurons (**A**, **B**, and **C**) that were determined under quiet conditions. The panels in the second to the sixth columns are the masked FLRFs determined under various levels of masker conditions. The masker level (from a to e) from the second to the sixth column increased monotonically, as shown at the right bottom corner of each panel (in dB SPL). Panel **D** indicates the responses of these neurons to the masker alone. Panels **E**, **F**, and **G** represent the effects of preceding noise on MT, FLRF area, and CF, respectively.

The FLRFs of 42 AI neurons were measured at four or five preceding noise levels. Figure 2 shows the FLRFs of three representative neurons determined under control (quiet) conditions and under conditions with preceding noise at different levels. Although the CFs and the frequency tuning ranges of these neurons in control conditions were quiet different (Figure 2A-1, B-1, and C-1), the FLRF data in Figure 2 demonstrated that preceding noise had monotonic suppressive effects on the frequency tuning of the neurons with increasing noise level. This can be seen from the monotonic increase in MT shift and the monotonic reduction in frequency tuning range (Figure 2 rows 1, 2, and 3; Figure 2E and F). The impact of preceding noise on the CF of the three neurons was not as apparent as the MT shift or the area change of FLRF (Figure 2G vs. Figure 2E and F). The CF shift by preceding noise was less than 0.25 octaves for neurons B and C, and the CF shift for neuron A was similar to neuron B and C except when tested at 80 dB SPL (level e) preceding noise condition. Interestingly, the responses of neuron A to the preceding noise alone at levels c, d, and e were similar (Figure 2D, filled circle); however, the masking effect of preceding noise on the MT and the FLRF area of neuron A was monotonically increased with increasing noise level (Figure 2E and F, filled circle).

**Figure 3:**
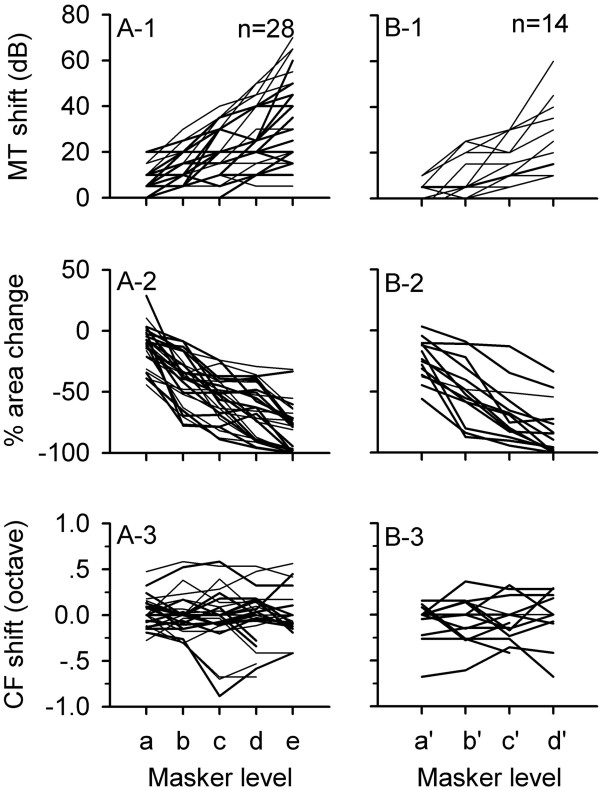
**Population data showing level-dependent modulation of frequency tuning by a preceding noise (masker).** The left column shows the data of 28 neurons whose FLRFs were determined at five levels of masker conditions, and the right column shows the data of 14 neurons whose FLRFs were determined at four levels of masker conditions.

The effects of preceding noise level on the FLRF for the population of the 42 neurons are shown in Figure 3. To normalize the masker level difference among neurons, the masker levels were placed into difference categories (Figure 3). When five levels of preceding noise were used, the levels were placed into category a, b, c, d, and e, respectively. Similarly, when four levels of preceding noise were used, the levels were placed into category a’, b’, c’, and d’, respectively. The relationships among different masker levels are the followings: a < b < c < d < e; a’ < b’ < c’ < d’. Generally, when the ISI was fixed, both the MT shift and the area change of FLRF were increased with increasing masker level. For the 28 neuron whose FLRFs were determined at five levels of noise, a repeated measure one-way ANOVA showed that a higher level of preceding noise induced a larger MT shift (Figure 3A-1, df = 4, F = 67.516, *p* < 0.001; LSD comparison at any two levels, *p* < 0.001) and a bigger area change of FLRFs (Figure 3A-2, df = 4, F = 113.259, *p* < 0.001; LSD comparison at any two levels, *p* < 0.001). Similarly, for the 14 neurons whose FLRFs were determined at four levels of noise, repeated measure one-way ANOVA showed that, the higher the noise level, the greater the effect on the MT shift (Figure 3B-1, df = 3, F = 30.720, *p* < 0.001; LSD comparison at any two levels, p < 0.005) and on the area change of FLRF (Figure 3B-2, df = 3, F = 58.189, p < 0.001; LSD comparison at any two levels, *p* < 0.001). However, the effect of noise level on CF shift varied among neurons (Figure 3A-3 and B-3), and the CF shift of 52.4% (22/42) of neurons were within 0.25 octaves under the noise levels tested. The CFs of the other 20 neurons (20/42, 47.6%) were significantly changed (>0.25 octaves) by preceding noise under at least one level of noise condition tested. The CF of the 42 neurons distributed in the range of 8.0 to 36.0 kHz, and no significant differences were found in the range of CF distributions between neurons with a significant CF shift and neurons without a significant CF shift. Repeated measure one-way ANOVA showed that the mean CF shift for the population neurons did not change significantly with the change of noise level (for the 28 neurons, df = 4, F = 0.266, *p* > 0.05; for the 14 neurons, df = 3, F = 0.358, *p* > 0.05).

**Figure 4:**
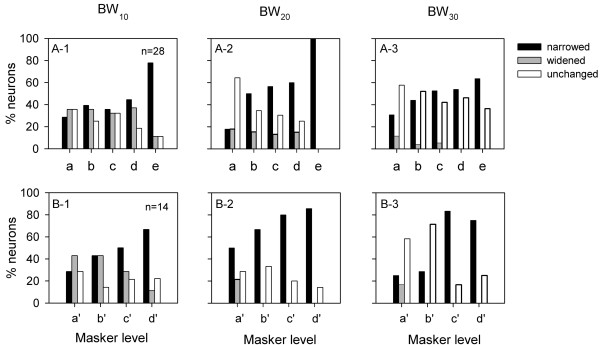
**Population data showing the percentage of neurons that fell into different categories of bandwidth change of FLRF.** BW_10_, BW_20_, and BW_30_ are the FLRF bandwidth at 10 dB, 20 dB, and 30 dB above MT, respectively. The changes of bandwidth were assigned into three categories: narrowed, widened, and unchanged. Row 1 shows the data of 28 neurons whose FLRFs were determined at five levels of masker conditions, and row 2 shows data of 14 neurons whose FLRFs were determined at four level of masker conditions. The relationships among different masker levels are the following: a < b < c < d < e; a’ < b’ < c’ < d’.

Preceding noise also affected the bandwidth of FLRF of most AI neurons tested. For each AI neuron, we analyzed the change of the FLRF bandwidth at n dB above MT (BWn) by comparing the BWn of the masked FLRF with the BWn of the control FLRF. However, we did not find a clear relationship between the percent change of the BWn and the level of preceding noise. Therefore, we categorized the effect of the preceding noise on BWn as follows: (1) widened, i.e., the change of BWn was greater than 20%, (2) narrowed, i.e., the change of BWn was less than −20%, (3) unchanged, i.e., the change of BWn was between −20% and 20%. Figure 4 shows the percentage of neurons that were associated with each respective category of bandwidth change under the conditions of various preceding noise levels. At a low level of noise, the percentage of neurons with a narrowed BW_10_ is similar to the percentage of the neurons with a widened BW_10_ (Figure 4A-1 and B-1); however, the percentage of neurons with a narrowed bandwidth is generally greater than the percentage of neurons with a widened bandwidth at BW_20_ and BW_30_ (Figure 4A-2, B-2, A-3 and B-3). Moreover, for most of the noise levels tested, the percentage of neurons with an unchanged bandwidth was greater at BW_30_ than that at BW_10_. This result suggests that the tip of the FLRF of an AI neuron has a greater chance to be impacted than the upper portion of FLRF under preceding noise conditions. Of the 42 neurons whose FLRFs were measured under preceding noise conditions, 20 neurons (47.6%) exhibited a narrowed BWn (for each neuron, at least one of the BWn was narrowed, and the other BWn was not changed); 7 (16.7%) showed a widened BWn (for each neuron, at least one of the BWn was widened, and the other BWn was unchanged); and 15 neurons (35.7%) exhibited a mixed effect of preceding noise on BWn (for each neuron, the bandwidths were narrowed at one BWn, and widened at another BWn).

### The effect of preceding noise on frequency tuning was time-dependent

**Figure 5:**
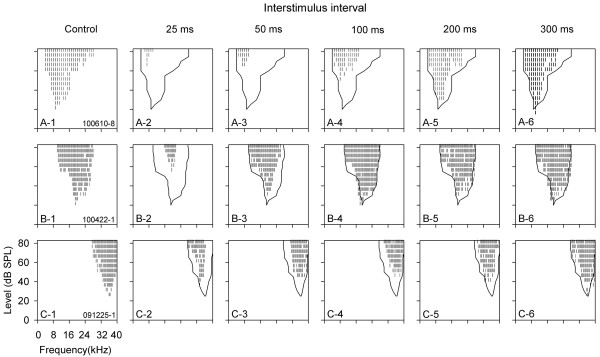
**The FLRFs of three example neurons showing that the modulation of frequency tuning by preceding noise was temporal-dependent.** Panels in each row are the data from one neuron. Panels in column 1 show the control FLRF, and panels in columns 2 to 6 show the masked FLRFs determined at various ISIs. The masker was a white noise at 50 dB SPL for the three neurons. The ISIs for conditions in each column are shown (in ms) on the top of the panels in row 1.

**Figure 6:**
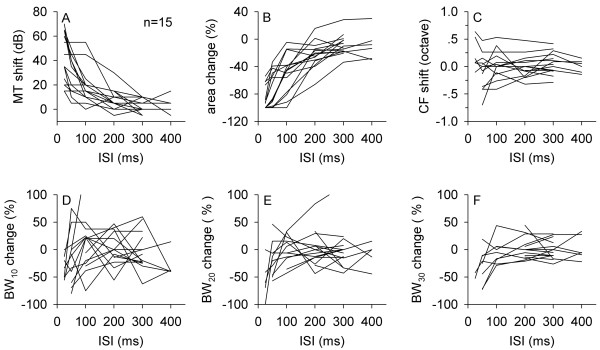
**Population data showing the time-dependent modulation of frequency tuning by preceding noise (masker).** Each line in the drawings depicts the data from one neuron including (**A**) the effect of masker on MT, (**B**) the percentage of area change of FLRF, (**C**) the CF shift, (**D**) the percent BW_10_ change, (**E**) the percent BW_20_ change, and (**F**) the percent BW_30_ change.

We examined the temporal effect of preceding noise on frequency tuning in 15 neurons by varying the ISI between 25 and 400 ms. The FLRFs of three example neurons with various CF under certain ISI conditions are shown in Figure 5. Although the impact of a preceding noise on the FLRFs varied among neurons, the data demonstrated that the suppressive effect of a preceding noise on the extent of FLRFs was greater at a shorter ISI as compared to a longer ISI (Figure 5, panels within rows 1, 2, and 3). The population data related to the temporal effect of a preceding noise on FLRFs are shown in Figure 6. The impact of noise was evident at short ISI, but was weakened with increasing ISI on the MT shift (Figure 6A), the area change of FLRF (Figure 6B), and the CF shift (Figure 6C). For eight of the 15 neurons, the noise presented at any ISI did not significantly affect their CFs (not more than 0.25 octaves; Figure 6C). Among the seven neurons whose CFs were affected by the noise, three neurons returned to their control CFs at 300 ms ISI. The trend of FLRF bandwidth change of the 15 neurons was not as apparent as the MT shift or the area change of FLRF with increasing ISI (Figure 6D, E, and F). Among the 15 neurons that were examined, the MT of 11 neurons and the FLRF area of 13 neurons recovered to similar levels as the control conditions at 300 ms ISI. However, the BW_10_ of 14 neurons, the BW_20_ of six neurons, and the BW_30_ of six neurons did not return to the control values at 300 ms ISI. These data suggested that the impact of a preceding noise on the tip of FLRFs lasted for a longer duration than that on the middle part of the FLRFs.

**Figure 7:**
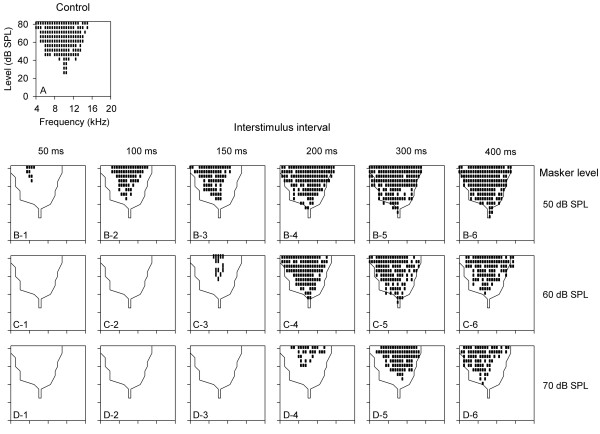
**The FLRFs of a neuron showing that the modulation of frequency tuning by preceding noise was temporal- and level-dependent.** Panel **A** depicts the control FLRF. The other panels in each row show the masked FLRFs determined at various ISI and masker level conditions. The panels in each column from rows two to four represent the masked FLRFs determined at a fixed ISI (numbers shown on the top panels of row 2) and under various masker level conditions. The numbers on the right to column six indicate the masker level for that row, i.e., 50 dB SPL (**B**), 60 dB SPL (**C**), and 70 dB SPL (**D**).

The temporal effect of the preceding noise on FLRF of AI neurons was also related to the level of noise. The FLRFs of a neuron that were determined under various noise levels and ISI conditions are shown in Figure 7. At a fixed level of noise, the influence of noise on FLRF decreased with increasing ISI (Figure 7, data shown in panels within rows 2, 3, and 4), and the impact of noise on FLRF at a fixed ISI increased as the level of noise increased (Figure 7, data in panels within each column of rows 2 − 4). The 70 dB SPL noise completely eliminated the responses within the control FLRF at 150 ms ISI, whereas the 50 dB SPL noise only partially suppressed the responses in the control FLRF at 50 ms ISI (Figure 7B-1 vs. Figure 7D-3). The effect of 50 dB SPL noise on FLRF diminished at 400 ms ISI, while the impact of 70 dB SPL noise on FLRF was still evident at this ISI (Figure 7B-6 vs. Figure 7D-6). These data demonstrate that the impact of a higher level of preceding noise on the FLRF lasted for a longer period of time compared with a lower level of preceding noise.

## Discussion

The detection and discrimination of sound frequency is necessary in order to perceive a sound signal for both humans and animals. Noise is ubiquitous in daily environment; however, how the auditory cortical neurons represent sound frequency information in a noisy environment is still not fully understood. The present study was designed to examine how the frequency tuning of AI neurons was affected by a preceding noise. In this study we found that the excitatory frequency receptive field of rat AI neurons was dynamically modulated by a varying preceding noise, and this modulation was dependent on the noise level and the temporal relationship between the noise and the probe.

The change of frequency tuning by a preceding noise was shown in both the MT shift and the change in the extent of FLRF. As the preceding noise level was increased, the response threshold of an AI neuron was monotonically elevated, and the extent of FLRF was monotonically reduced. This result is consistent with the trends found in our previous study using a tone-tone forward masking paradigm [29]. Studies in the inferior colliculus [31,32] also showed that an effective forward masker elevated the MT of inferior collicular neurons. Previous psycophysical studies have shown that the detection threshold of a target sound was elevated when preceded by a forward masker [3]. The neurophysiological results found in the present and previous studies have provided neural correlates for the psychophysical result. It should be notenoted that the increase in the percentage of area change of FLRF is not solely caused by the MT shift. The change of the bandwidth of FLRF also contributes to the change of the extent of FLRF. The FLRF bandwidths of many neurons were narrowed following a preceding noise. In contrast, the FLRF areas of other neurons were reduced despite of a widening effect at the tip of FLRF. From the population coding perspective, an effective preceding noise reduces the number of neurons tuned to the probe stimulus when the probe sound is at low and intermediate levels, and consequently deteriorates the detection and discrimination of the frequency of the probe sound.

Sound frequency is tonotopically represented across the central auditory system. For instance, in quiet conditions the tonotopic maps were found in the auditory cortex of cat [33], rat [30], mouse [34,35], marmoset [36] and chinchilla [37]. The present study showed that the CFs of more than half of the tested neurons were not significantly changed by a preceding noise, suggesting that the frequency preference of these neurons were resistant to a change under conditions of noise interference. The relative stability of the CFs of these tested neurons suggests that the tonotopic maps determined under quiet conditions might be partially maintained under preceding noise conditions. For a few neurons, their CFs were shifted significantly by preceding noise (Figure 3A-3). AI neurons receive inputs from multiple sources; preceding noise might differentially affect a portion of these inputs that contribute to the responses at or near the CF of an AI neuron and therefore resulted a great CF shift. Another interesting aspect of this study is that preceding noise narrowed the tip of FLRF in 47.6% of the neurons that were tested. This narrowed FLRF increases the selectivity of neurons to sound frequency, and is useful for frequency discrimination. Consequently, humans and animals could still maintain the ability to detect and discriminate sound frequency in a noisy environment, but this ability would likely be improved in quiet conditions.

Our results demonstrated that the extent of the masked excitatory FLRF is dependent on the interactions between the masker and the probe stimuli. The suppressive effect of preceding noise on the frequency tuning of AI neurons is partially dependent on the noise level. Compared to a lower level of preceding noise, a higher level of preceding noise induced a stronger suppression on frequency tuning of neurons to low and intermediate levels of probe stimuli, resulting in a more focused excitatory FLRF responding to high level of probe stimuli. These findings suggest a competitive interaction between the probe response and the suppression induced by the masker [18]. Previous studies in cat found that the monotonicity of suppressive effect of a forward masker on level tuning is closely related to the monotonicity of the rate-level functions of an AI neuron [16,17]. In the present study, we have determined the rate-level functions of AI neurons to preceding noise alone; however, we recorded the responses to each stimulus condition in the FLRFs of an AI neuron using only two presentations of the probe stimuli. Consequently, it is difficult to determine the relationship between the monotonicity of the noise rate-level functions of AI neurons and the monotonicity of the suppressive effects of preceding noise on the FLRFs.

Previous psychophysical experiments have shown that the forward masking effect was stronger at a shorter masker-probe delay and was weaker at a longer delay [38]. In the present study, the suppressive effects by a preceding noise on frequency tuning of AI neurons were decreased with increasing ISI, and lasted for several hundred milliseconds. The time course of suppression by preceding noise on excitatory frequency tuning determined in this study was similar to that found in previous studies using tone-tone masking paradigm to study contextual modualtion of excitatory frequency tuning of AI neurons in rat [29], to examine the inhibitiory frequency tuning of AI neurons in cat [27], and to determine the contextual modulation of level tuning [21] and spatial tuning [25] in cat. After a noise stimulus occurs, the responses in the FLRF to a probe at a higher level recovered earlier than that at a lower level. We also found that the time course of the suppressive effect is related to the level of the masker, i.e., a noise stimulus at a higher level induced a longer duration of suppression than a noise stimulus at a lower level. In the marmoset auditory cortex, a longer duration masker induced a long-lasting (>1 s) effect on the responses to probe stimuli [7]. Therefore, the time course of cortical interaction of sounds in sequence depends on the stimulus parameters of both the preceding sound and the following sound.

Our study showed that preceding noise induced a suppressive effect on frequency tuning of rat AI neurons. However, the amount of cortical contribution to the suppressive effect is unclear. Previous studies in the inferior colliculus of marmoset and mouse demonstrated that a forward masker elevated the response threshold [32] and sharpened the frequency receptive field [31] of inferior collicular neurons. Numerous studies have demonstrated the existence of GABAergic and glycinergic input on to the inferior collicular neurons [e.g., [39]]. These results suggest that subcortical nuclei might contribute to the suppressive effects observed in the present study. Studies in the auditory cortex of rat [8,29,40], cat [21], and marmoset [7] indicate that the time course of suppression induced by an effective forward masker lasted for several hundred milliseconds or longer. In contrast, the inhibitory conductance induced by a forward masker in the rat auditory cortex rarely lasted longer than 50 − 100 ms [8,40], therefore, the postsynaptic suppression cannot account fully for the long time course of suppression. Zador and his colleagues proposed that presynaptic depression contributes to the long lasting suppression. It has been shown that pentobarbital prolonged the inhibitory conductance and enhanced the suppression induced by a sound stimulus [8,41]. Therefore, the suppression on the frequency tuning observed in rat AI neurons in the present study likely originates from multiple sources including subcortical contribution, pentobarbital anesthesia as well as postsynaptic suppression and presynaptic depression in the auditory cortex.

## Conclusions

The results in the present study have demonstrated that the cortical representation of sound frequency is dynamically modulated by contextual noise, and that the degree of modulation is dependent on the acoustical parameters of the preceding noise and the probe sound. Moreover, there exist cortical neurons whose characteristic frequencies were resistant to the contextual noise interference.

## Abbreviations

AI, Primary auditory cortex; BWn, Bandwidth at n dB above MT; CF, Characteristic frequency; FLRF, Frequency-level receptive field; ISI, Interstimulus interval; MT, Minimum threshold.

## Competing interests

The authors declare that they have no competing interests.

## Authors’ contributions

YP contributed to the acquisition of data, analysis and interpretation of the data. PX and JH contributed to the analysis of the data. JZ and XS contributed to the design of the experiment and the writing of the manuscript. All authors read and approved the final manuscript.
